# The intestinal fatty acid-binding protein as a marker for intestinal damage in gastroschisis

**DOI:** 10.1371/journal.pone.0210797

**Published:** 2019-01-14

**Authors:** Alena Kokesova, Stepan Coufal, Barbora Frybova, Miloslav Kverka, Michal Rygl

**Affiliations:** 1 Department of Pediatric Surgery, Charles University in Prague, 2nd Faculty of Medicine, University Hospital Motol in Prague, Prague, Czech Republic; 2 The Czech Academy of Sciences, Institute of Microbiology, Prague, Czech Republic; 3 The Czech Academy of Sciences, Institute of Experimental Medicine, Prague, Czech Republic; Beth Israel Deaconess Medical Center, Harvard Medical School, UNITED STATES

## Abstract

**Background/Purpose:**

We analyzed the capacity of urinary Intestinal fatty acid-binding protein (I-FABP) to quantify the degree of mucosal injury in neonates with gastroschisis (GS) and to predict the speed of their clinical recovery after surgery.

**Methods:**

In this prospective study, we collected urine during the first 48h after surgery from neonates operated between 2012 and 2015 for GS. Neonates with surgery that did not include gut mucosa served as controls for simple GS and neonates with surgery for intestinal atresia served as control for complex GS patients. The I-FABP levels were analyzed by ELISA.

**Results:**

Urinary I-FABP after the surgery is significantly higher in GS newborns than in control group; I-FABP in complex GS is higher than in simple GS. I-FABP can predict subsequent operation for ileus in patients with complex GS. Both ways of abdominal wall closure (i.e. primary closure and stepwise reconstruction) led to similar levels of I-FABP. None of the static I-FABP values was useful for the outcome prediction. The steep decrease in I-FABP after the surgery is associated with faster recovery, but it cannot predict early start of minimal enteral feeding, full enteral feeding or length of hospitalization.

**Conclusion:**

Urinary I-FABP reflects the mucosal damage in gastroschisis but it has only a limited predictive value for patients’ outcome.

## Introduction

Gastroschisis (GS) is a congenital anomaly of the abdominal wall, which results in extrusion of abdominal viscera from the abdominal cavity. The prevalence of GS is increasing; currently reaching 4.9 per 10,000 live births [1]. Although survival in GS exceeds 90%, some babies experience significant morbidity, which is largely determined by the severity of prenatal and postnatal bowel injury [2].

It is still unclear how the structural changes correlate with malabsorption and small bowel dysmotility in GS and therefore with patient outcomes [3]. The extensive injury of intestinal mucosa could be a major source of complications both during the surgery and during the postoperative period and may have a major impact on patient recovery. A biomarker capable of quantifying the condition of intestinal mucosa after closure of the abdominal wall and during the acute post-surgery period is needed in clinical practice. I-FABP (Intestinal fatty acid-binding protein) is present in the cytoplasm of mature enterocytes in the small and large intestine and is released as soon as the cell membrane integrity is compromised, thus reflecting the extent of gut damage. It is used as a biomarker of mucosal injury and other diseases affecting the intestine [4]. Serum I-FABP correlates with the severity of villous atrophy in coeliac disease and with Crohn's disease activity [5, 6]. It is increased after abdominal surgery or infection and can be used as a biomarker of acute gut mucosa damage in necrotizing enterocolitis (NEC) or distinguish it from neonatal sepsis [7–9].

The aim of this prospective study is to find out if I-FABP could be used as a biomarker for mucosal injury in neonates after the GS surgery and if it can predict the speed of their clinical recovery.

## Materials and methods

### Population under study

Thirty-two patients with GS were recruited from the patients admitted to the Neonatal Intensive Care Unit, Department of Pediatric Surgery of University Hospital Motol, Prague, between 2012 and 2015. Two patients were excluded from the study because they died within the first 48 hours of life without passing any urine. One died of fulminant septic shock and one died of multiorgan failure resulting from prenatally acquired massive intestinal necrosis. Their samples and follow up were therefore not complete for later analysis. Out of our study group, 26 were born in our center and 4 were born in peripheral hospitals and transferred to our department immediately after delivery. All patients were operated within first 6 hours of life regardless of the place of birth.

Categorization of GS as simple or complex was based on the absence or presence of intestinal atresia, stenosis, perforation, necrosis or volvulus at birth, as suggested by Molik et al. [10]. All of our complex GS patients had small intestinal atresia, in 2 patients was intestinal atresia found during second revision, so these two individuals were reclassified as complex GS.

All subjects of this study were operated under general anesthesia in the operating theatre. A primary operative abdominal wall repair was attempted in all our patients. Primary closure was performed by an interrupted absorbable suture with attention to preserving the umbilicus. Gore-Tex silo (1mm Dual Gore-Tex patch) was used when primary closure was not possible. The decision was made by attending surgeon when viscera could not be primarily reduced without danger of impeding venous return, increase of peak inspiratory pressures and increase of abdominal pressure. The final closure and silo removal was performed at 18 (12–29) (median (range)) day of life.

Total parenteral nutrition was started within 24 hours of life via central venous catheter and performed in compliance with the current recommendation [11]. The nutritional regimen for all patients included mixture of amino acids (Primene 10%, Baxter Czech, Prague, Czech Republic), fat (Smoflipid 20%, Fresenius Kabi AB, Uppsala, Sweden) and dextrose, supplemented with the vitamins and trace-elements (Soluvit ^®^ N, Vitalipid^®^ N Infant, Peditrace^®^, Fresenius Kabi AB, Uppsala, Sweden).

Enteral feeding was introduced once postoperative ileus had been resolved and the decision was made by attending neonatologist. Neonates received mother’s milk or bank milk continuously, via a nasogastric tube. Feeding was increased gradually according to tolerance and measurement of gastric residua. After initiation of feeding we followed minimal enteral feeding protocol as follows. Breast milk was given via nasogastric tube continuously, starting at 0.3–0.5 ml/kg/h. If it was well tolerated, the feeding was increased by 7–12 ml/kg daily. If neonate didn’t tolerate the daily increase, the dose was restored to the last tolerated amount or stopped entirely.

To exclude the confounding factor of surgery, we established a different control group for either type of GS—newborns who underwent surgery without intestinal mucosa disruption (S1 Table) served as controls for simple GS. All of our complex GS patients had small intestinal atresia, so control group for complex GS sustained from patients operated for small intestinal atresia, who underwent as well as complex GS patients resection of atretic part of the intestine and then end-to end anastomosis. There were 12 newborns in the control group for simple GS and 6 newborns in the control group for complex GS.

To analyze the capacity of urinary I-FABP to predict the speed of the postoperative recovery, we used clinical data from patients’ health records and chose time to minimal enteral feeding of 20 ml/kg/day (MEF), time to full enteral feeding (FEF) and length of hospitalization (LOH). The median number of days was a cut off. Therefore, we defined "early" MEF as less than 13 days, "early" FEF as less than 19 days and "short" LOH as less than 31 days. The detailed demographics of studied population is summarized in Table 1.

**Table 1:** The demographic data of the study group.

	GS simple (n = 25)	Control (n = 12)	P	GS complex (n = 5)	Control (n = 6)	P
**Delivered by C-section, n (%)**	10 (40.0)	3 (25)	0.46	3 (60)	2 (33.3)	0.58
**Male, n (%)**	13 (52.0)	8 (66.6)	0.49	2 (40)	3 (50)	1.0
**Median gestation age in weeks (range)**	36 (32–38)	39 (33–41)	< 0.01	34 (33–37)	39 (37–40)	< 0.01
**Median birth weight in grams (range)**	2400 (1490–3650)	2925 (1800–4200)	< 0.05	2400 (1475–2790)	3160 (2510–3445)	< 0.01
**Prenatal diagnosis, n (%)**	22 (88.0)	-	-	5 (100)	-	-
**Treated with primary closure, n (%)**	20 (80.0)	-	-	4 (80)	-	-
**Treated with stepwise reconstruction, n (%)**	5 (20.0)	-	-	1 (20)	-	-
**Median hospital stay in days (range)**	26.0 (10–83)	6.5 (3–25)	< 0.001	72.0 (28–93)	18.0 (10–28)	< 0.01

The study was approved by the Ethics committee of the University Hospital Motol, and written informed consent was obtained from parents of all children included in this study.

### Sample collection and processing

Urine samples were collected for 48 hours in 6 hours’ intervals, starting during the first 6h after the surgery. Samples were collected from urine bag connected to an indwelling catheter. The urinary creatinine was measured in each sample in our hospital biochemistry laboratory immediately after sampling and samples for I-FABP analysis were frozen at -20°C until the analysis.

### Enzyme-Linked Immunosorbent Assay (ELISA)

Urinary I‐FABP was measured by Human I-FABP ELISA (Hycult Biotech, Uden, The Netherlands). The assay was performed according to the manufacturer’s instruction. To eliminate fluctuation in urine excretion, the urinary I-FABP was normalized to urinary creatinine and is presented as pg/nmol of creatinine.

### Statistical analysis

The continuous variables were tested for normality by D'Agostino-Pearson normality test. The differences between GS and control group were analyzed either by Mann-Whitney test (continuous variables) or by Fisher's exact test (dichotomous variables). I‐FABP levels in complex and simple GS were compared with each other and with these in controls using Kruskal-Wallis test with Dunn's post test. I-FABP was correlated with the time to MEF, FEF and with LOH using Spearman's rank correlation coefficient (r). Continuous variables are presented as median (range), dichotomous variables as the number of cases (percentages), and p values < 0.05 were considered statistically significant.

Correlation matrix was created using Spearman's rank correlation coefficient (r), and p values were divided by the number of analyzed values to prevent the type I error (false positivity). Predictive value of the I-FABP for the MEF, FEF and LOH was analyzed using receiver operating characteristic (ROC) curves. Statistical analyses were performed using GraphPad Prism version 6.0 (GraphPad Software, San Diego, CA, USA). Ordinary least squares (OLS) regression was used to model the relationship between MEF, FEF and LOH and I-FABP levels at different time points. Regression analysis was performed in R (ver. 3.3.2; R Foundation for Statistical Computing, Vienna, Austria) and Akaike information criterion (AIC) was determined in the MASS package (ver. 7.3–45). Next, we performed both backward elimination and forward selection based on AIC to determine the best regression model.

## Results

**Fig 1:**
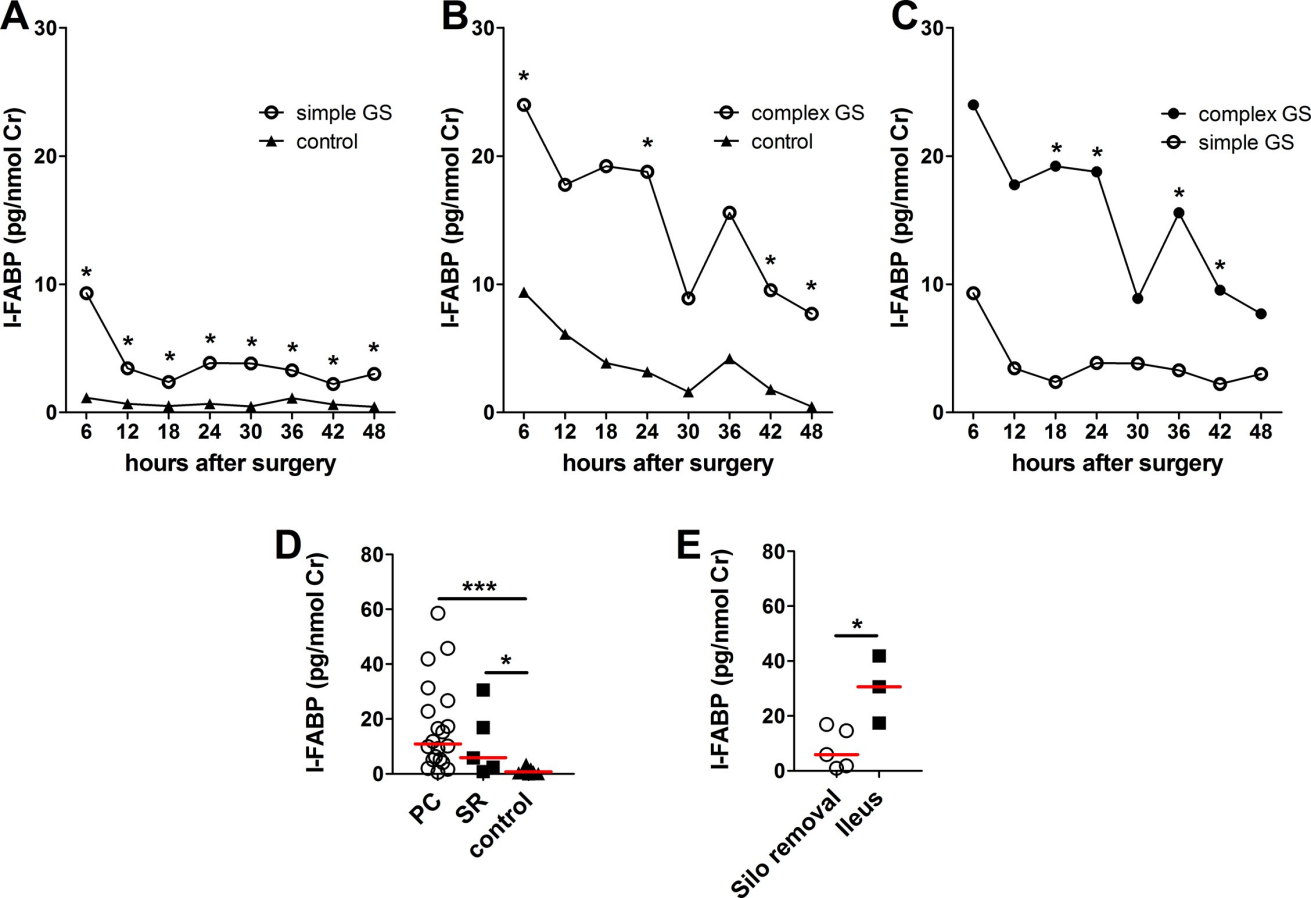
The analysis of urinary I-FABP. Urinary I-FABP after the surgery in simple GS (Fig 1A) and complex GS (Fig 1B) vs. controls. Comparison of I-FABP between simple and complex GS after the surgery (Fig 1C). I-FABP in groups treated with stepwise reconstruction (SR) or primary closure (PC) and in controls (Fig 1D). Urinary I-FABP during the first 6 hours after surgery (6h) in complex GS patients who will be later operated for mechanical ileus and in those operated only for silo removal (Fig 1E). Median *p<0.05, **p<0.01 and *** p<0.001.

Simple and complex GS patients have higher urinary I-FABP after the surgery than control subjects (Fig 1A and 1B). The I-FABP dynamics in simple and complex GS don’t differ. In both cases, I-FABP levels reach maximum in the first 6h after surgery (9.29 (0.59–58.56) pg/nmol for simple GS, 23.99 (16.59–41.90) pg/nmol for complex GS). Interestingly, I-FABP peaks 36 hours after the surgery in both patients with complex GS (15.57 (28.73–8.03) pg/nmol) and in controls for complex GS (4.20 (0.31–10.89) pg/nmol). The level of urinary I-FABP in controls for simple GS is generally low and without a distinct peak; in first 6 hours reaches 1.15 (0.06–3.41) pg/nmol.

In the first 48h after the surgery, the levels of I-FABP in patients with complex GS are higher than in patients with simple GS (Fig 1C). We did not find significant differences in I-FABP levels between patients treated with primary closure (PC) and stepwise reconstruction (SR), but I-FABP in both these groups was significantly higher than in controls (Fig 1D).

Urinary I-FABP during the first 6 hours after surgery is significantly higher in complex GS patients who will be later operated for mechanical ileus than in those operated only for silo removal (Fig 1E).

There is a clear difference in the outcome between complex and simple GS. Patients with complex GS have received FEF significantly later (median 59 vs. 17 days, p = 0.0055) and have significantly longer LOH than patients with simple GS (median 72 vs. 26 days, p = 0.0120). We used a linear regression model to analyze at which time point the urinary I-FABP has the highest capacity as a predictive biomarker for clinical outcome (MEF, FEF and LOH). Due to the clear differences in I-FABP dynamics, we included the changes in I-FABP as additional variables. None of the I-FABP levels measured at 6-hour intervals was a suitable predictor for the outcomes. However, three changes in I-FABP levels in time (decrease between 12 and 18 hours, decrease between 12 and 24 hours and increase between 24 and 30 hours) were found to be exceptionally good predictors (Table 2).

**Table 2:** Regression analysis outcome of the suitable models.

Model	12h-18h		12h-24h		24h-30h	
	Effect±SE	Adjusted R^2^	Effect±SE	Adjusted R^2^	Effect±SE	Adjusted R^2^
**MEF**	-2.40±0.42	0.71***	-1.93±0.4014	0.63***	1.08±0.26	0.55**
**FEF**	-2.35±0.60	0.53**	-1.88±0.5387	0.46**	1.05±0.34	0.40**
**LOH**	-2.00±0.68	0.37*	-1.55±0.6109	0.29*	1.00±0.34	0.37*

*p<0.05

** p<0.01

*** p<0.001

**Fig 2:**
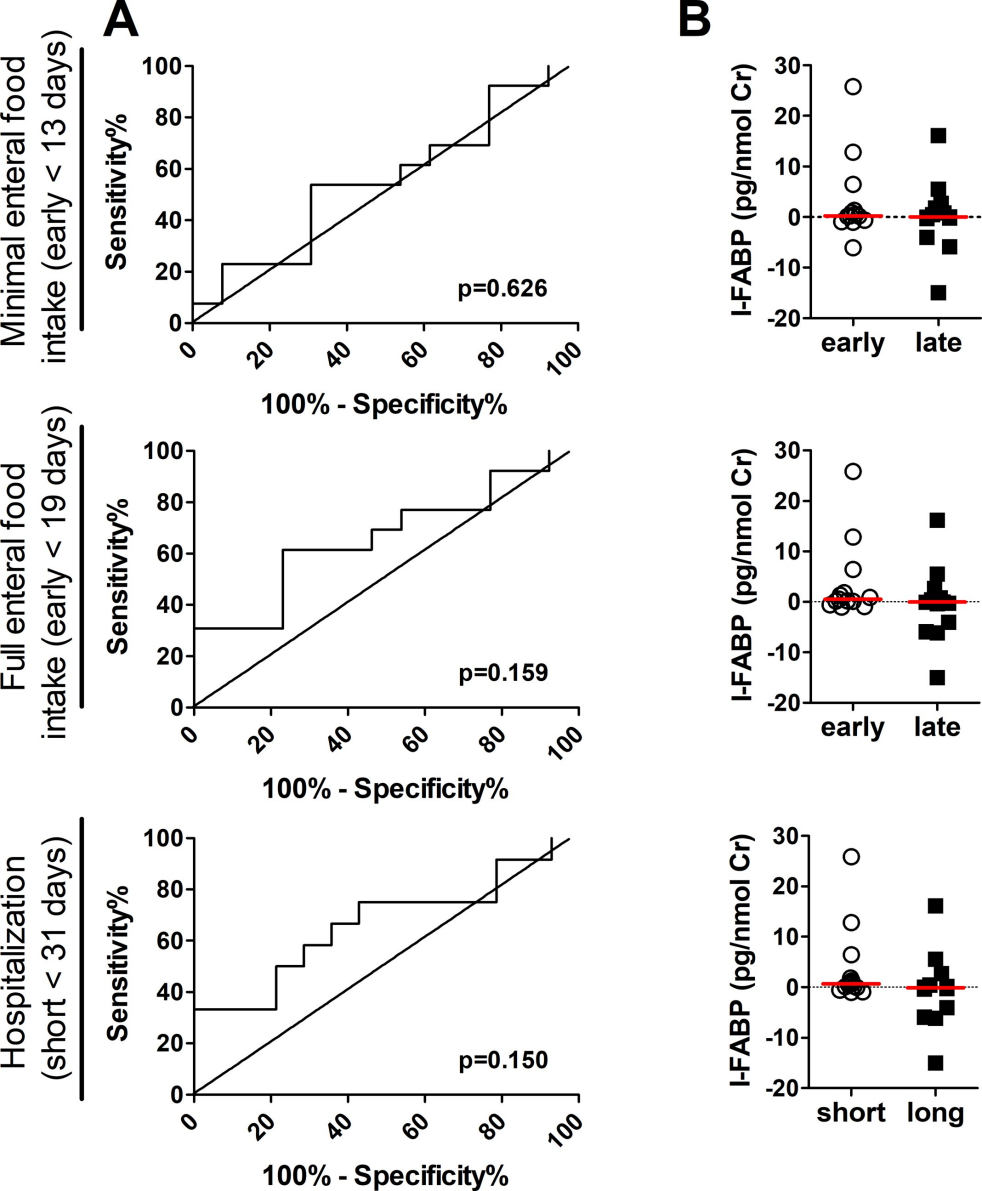
The analysis of predictive capacity of I-FABP for clinical outcome. The decrease in urinary I-FABP between 12 and 18h after the surgery (ΔI-FABP 12-18h) does not distinguish between early and late start of minimal/full enteral feeding or short and long hospitalization as measured by ROC curve analysis (Fig 2A) or conventional statistics (Fig 2B).

All these models provide an equivalent fit for LOH and FEF (Δ AIC < 3), but 12h-18h is equivalent to 12-24h (Δ AIC = 3) and gives slightly better fit than 24-30h (Δ AIC = 6). This was, however, not supported by ROC curve analysis, in which the decrease in I-FABP between 12-18h was not capable of predicting early MEF/FEF or short LOH, as defined for our dataset (Fig 2A). The levels of I-FABP at the time of surgery do not predict multiple surgeries, as analyzed by ROC curve analysis (S1 Fig).

To analyze the correlation of individual values and demographic characteristics of GS patients, we constructed a correlation matrix (S2 Table). We found a clear correlation among the levels of I-FABP at different time points, among all three recovery characteristics and between gestation length and birth weight. Another well-established connection is that patients with multiple surgeries are less likely to be operated with primary closure and have longer LOH. The raw data with appropriate metadata are in S3 Table.

## Discussion

Disruption of intestinal mucosa causes major complications in patients with GS. The extent of this injury and its capacity to predict patient’s recovery has not yet been sufficiently analyzed. Our study showed that I-FABP can serve as a biomarker for the gut mucosa damage after the closure of abdominal wall in GS.

I-FABP is a small cytoplasmic protein localized in epithelial cells of the small intestine [12], which is released into the circulation after enterocyte damage [13] and quickly passes into urine. Therefore, urinary I-FABP could be used as a non-invasive biomarker of acute gut mucosa damage in spontaneous and surgery-related necrotizing enterocolitis [7, 9, 14–16]. Since gut mucosa damage is a typical pathological feature of GS, we studied if urinary I-FABP could be also used as a biomarker to predict a patient's outcome.

We found that I-FABP is higher in patients with complex GS as compared to simple GS, which is consistent with significantly more severe mucosal damage in complex GS. However, traumatization to the gut during intestinal incision leads to a quick increase in plasmatic I-FABP as well [8], so it is unclear if this is an effect of more extensive damage during complex GS or just an effect of extensive surgery. All complex GS patients in this study had intestinal atresia, so they underwent intestinal wall incision, intestinal resection and intestinal wall suture. To control for the confounding factor of surgery and general anesthesia, we established a different control group for each type of GS—newborns who underwent surgery without intestinal mucosa disruption served as controls for simple GS and those operated for intestinal atresia with the same surgical technique were selected as controls for complex GS. These control groups were well matched for all characteristics except for those inherently associated with GS itself (gestational age and birth weight) and GS severity (hospital stay). We were not able to find any relevant information on the effect of gestational age and birth weight on I-FABP in the literature. None of these factors, however, correlated with I-FABP levels (S2 Table), so they do not seem to be crucial. Nevertheless, overall immaturity may influence the recovery regardless of gut damage, thus presenting a potential confounding factor. We found that while surgical damage to the gut mucosa increases the urinary I-FABP, its levels are significantly higher in both types of GS when compared to the relevant control group. Our results also showed that anesthesia, cold stress, volume therapy during surgery and general postoperative care do not influence I-FABP. Moreover, the I-FABP levels in controls for simple GS are not markedly higher than those in generally healthy newborns [7]. These results clearly show that damage to the gut mucosa in GS is not just a result of mucosal integrity disruption during surgery.

In this study, both ways of abdominal wall closure (i.e. primary closure and stepwise reconstruction) led to similar levels of I-FABP, suggesting that neither approach leads to more severe damage to the gut mucosa. In a recent meta-analysis, Kunz et al. compared short term outcomes of primary closure (PC) versus stepwise reconstruction (SR), finding that SR is associated with improved outcomes only if the method is selected randomly. Conversely, when the method is selected by the surgeon, PC is associated with improved outcomes [17]. This is probably caused by the fact that patients receiving silo are also more likely to be prone to worse clinical outcomes [18].

We found that while I-FABP quickly decreases in GS after the surgery, in patients with complex GS and controls with intestinal atresia, it increases again with a distinct peak at 30–36 hours after the surgery. This suggests that this delayed release of I-FABP after the small intestine surgery is either caused by protracted stricture of the circulation, which combines higher intestinal damage and delayed I-FABP release, or that it is a result of re-perfusion damage to the intestine [4]. This is in agreement with studies on animal models of GS, showing that increased intra-abdominal pressure leads to gut mucosa damage, possibly via oxidative stress and an increase in apoptotic activity of enterocytes [19, 20].

Interestingly, I-FABP is significantly higher in neonates with complex GS that were later operated for mechanical ileus compared to those operated just for silo removal, which further supports the hypothesis about continuous gut damage. These data, however, need to be interpreted with caution, because the number of neonates in both subgroups is low.

We found that the patient's recovery (MEF, FEF and LOH) is significantly faster in patients with simple GS than in those with complex GS. Several markers have been used to determine the patient outcome after GS surgery. Most of them focus on the sonographic findings on the fetal gut during prenatal examination. Dilated stomach of the fetus with gastroschisis is associated with higher neonatal death rate, volvulus, delayed enteral feeding and longer hospital stay postnatally [21]. Intraabdominal bowel dilation of multiple intestinal loops predicts not only earlier delivery, but also postnatal bowel complications in neonates with gastroschisis [22]. Not only the extent of the intraabdominal bowel dilation, but also its early appearance is associated with poor prognosis [23]. Prenatal bowel dilatation is associated with increased morbidity in patients with simple GS [24–26]. Measurement of the intraabdominal pressure (IAP) during surgery for gastroschisis may help to select optimal surgical technique and shorten the hospital stay [27]. Since we have just started with routine measurements of IAP during this study, we do not have data to correlate this potential biomarker with I-FABP levels.

We hypothesized that disruption of intestinal mucosal layer could be a major cause of complications in patients with GS and that the higher degree of gut mucosa damage predisposes to slower post-operative recovery. Since there are no guidelines for the division into early MEF/FEF and short LOH, we decided to use an unbiased approach and divide our dataset into two halves. Unfortunately, none of the measured levels of I-FABP could distinguish between these categories, although the decrease in I-FABP between 12 and 18h (or 12 and 24h) post-surgery explained well the variation of all three measured outcomes in regression analysis (Table 2).

We found that urinary I-FABP during the first 6 hours after surgery was significantly higher in complex GS patients who would be later operated for mechanical ileus than in those operated only for silo removal. There were, however, not enough patients with complex GS re-operated for ileus in our dataset to allow appropriate statistical analysis. The main advantage of single-center studies of rare diseases is the absence of variation between centers, but it comes at a cost of a small study population. This means that the statistical analysis is underpowered and notable to properly assess some important facets of the disease. This is the main reason why we could not compare individual surgery protocols and why we could define only the broadest disease stages (simple GS vs. complex GS) and basic procedures (PC vs. SR).

## Conclusions

Urinary I-FABP is a marker for intestinal mucosa damage in GS. Patients with complex GS have significantly higher levels of I-FABP and their recovery takes longer than in patients with simple GS. I-FABP fails to predict early MEF/FEF or shorter LOH, so it is not suitable for prediction of these parameters in clinical settings. Its capacity to predict subsequent operation for ileus in patients with complex GS needs to be interpreted with caution until a larger cohort of these patients is analyzed.

## Supporting information

S1 Fig The analysis of predictive capacity of I-FABP for multiple surgeries. (TIF)

S1 Table Type of surgery in controls for simple GS. (DOCX)

S2 Table Correlation matrix of individual values and demographic characteristics of GS patients. (XLSX)

S3 Table The raw data of studied GS and control patients. (XLSX)

## Abbreviations

GS: Gastroschisis
I-FABP: Intestinal fatty acid-binding protein
MEF: Minimal enteral feeding
FEF: Full enteral feeding
LOH: Length of hospitalization
PC: Primary closure
SR: Stepwise reconstruction
