# Supplementary material for: The intestinal fatty acid-binding protein as a marker for intestinal damage in gastroschisis
Source: PLoS One. 2019 Jan 14;14(1):e0210797. doi: 10.1371/journal.pone.0210797 (PMC6331122; doi:10.1371/journal.pone.0210797)
Supplement: S1 Table — (DOCX) [file pone.0210797.s002.docx]

| **Diagnosis** | **Number of patients** | **Type of surgery** |
| --- | --- | --- |
| Cleft lip | 3 | Correction surgery |
| Cystic lung disease | 1 | Pulmonary resection |
| Diaphragmatic hernia | 1 | Hernioplasty |
| Diaphragmatic relaxation | 1 | Plication surgery |
| Hydronephrosis | 1 | Heminephrectomy |
| Hydronephrosis | 1 | Pyeloplasty |
| Pylorostenosis | 1 | Pyloromyotomy |
| Sacrococcygeal teratoma | 2 | Extirpation |
| Thoracic hamartoma | 1 | Extirpation |
